# Revisiting social vulnerability analysis in Indonesia data

**DOI:** 10.1016/j.dib.2021.107743

**Published:** 2021-12-23

**Authors:** Robert Kurniawan, Bahrul Ilmi Nasution, Neli Agustina, Budi Yuniarto

**Affiliations:** a Department of Statistical Computing, Polytechnic Statistics STIS, Jakarta 13330, Indonesia; b Department of Statistics, Polytechnic Statistics STIS, Jakarta 13330, Indonesia; c Jakarta Smart City, Department of Communications, Informatics, and Statistics, Jakarta 10110, Indonesia

**Keywords:** Disaster management, Disaster mitigation, Fuzzy geographically weighted clustering, Multistage sampling, Social vulnerability

## Abstract

This paper presents the dataset about the social vulnerability in Indonesia. This dataset contains several dimensions which rely on previous studies. The data was compiled mainly from the 2017 National Socioeconomic Survey (SUSENAS) done by BPS-Statistics Indonesia. We utilize the weight to obtain the estimation based on multistage sampling. We also received additional information on population, the number, and population growth from the BPS-Statistics Indonesia's 2017 Population projection. Furthermore, we provide the distance matrix as the supplementary information and the number of populations to do the Fuzzy Geographically Weighted Clustering (FGWC). This data can be utilized to do further analysis of social vulnerability to promote disaster management. The data can be accessed further at https://raw.githubusercontent.com/bmlmcmc/naspaclust/main/data/sovi_data.csv.

## Specifications Table

**Table:** 

Subject	Geography
Specific subject area	Disaster management and risk reduction, social vulnerability
Type of data	Table
How data were acquired	The data was acquired from the 2017 National Socioeconomic survey from BPS-Statistics Indonesia and Indonesia 2013 Geospatial Map Instruments: Rstudio
Data format	Raw Analyzed Filtered
Parameters for data collection	We consider to use the district level data and match the existing data with the available districts in the maps
Description of data collection	We collect the raw data of the 2017 National Socioeconomic survey from BPS. Subsequently, we aggregated the data using the appropriate rules and used the weight to represent the sampling method. Moreover, we obtain the distance matrix from the data processing from the district-level map.
Data source location	2017 National Socioeconomic survey 2013 Indonesia geospatial map in district level
Data accessibility	With the article Repository name: naspaclust package GitHub Direct URL to data: Social vulnerability: https://raw.githubusercontent.com/bmlmcmc/naspaclust/main/data/sovi_data.csv Interdistrict distance matrix: https://raw.githubusercontent.com/bmlmcmc/naspaclust/main/data/distance.csv
Related research article	B.I. Nasution, R. Kurniawan, T.H. Siagian, A. Fudholi, Revisiting social vulnerability analysis in Indonesia: An optimized spatial fuzzy clustering approach, Int. J. Disaster Risk Reduct. 51 (2020) 101801. https://doi.org/10.1016/j.ijdrr.2020.101801.

## Value of the Data

• The dataset provides the development and disaster indicators from 511 districts in Indonesia and the distance matrix between districts.
• The dataset can be used to compare and evaluate the development of districts in Indonesia, followed by an elaboration in social vulnerability context as one of them.
• The availability of the dataset can help policymakers initiate responses to natural disasters by considering the regional developments and conditions.
• The dataset can identify the deeper regional development and hazards resilience for future studies, specifically using a spatial approach.
• The dataset can be combined with data from other study fields, such as public health and transportations, to obtain a deeper understanding of regional development in various contexts.

## 1 Data Description

Indonesia is one of the countries prone to various natural disasters, considering that geographically Indonesia is located in the Pacific Ring of Fire and is located at the meeting point of the world's three main tectonic plates [1]. Therefore, all districts in Indonesia are prone to natural disasters such as earthquakes, tsunamis, and volcanic eruptions. Furthermore, social vulnerability plays an important role in analyzing the impact of disaster, which refers to a community's susceptibility to the natural hazard damage, affecting its ability to recover [2]. Social vulnerability studies have emerged at the national level in Indonesia since Siagian et al. [3]. Furthermore, research by Nasution et al. [4] also analyzed social vulnerability by clustering districts in Indonesia using FGWC with Intelligent Firefly Algorithm (IFA). The method is available in an R package called naspaclust [5].

This study disseminates the dataset used in Nasution et al.'s research, entitled “Revisiting Social Vulnerability Analysis in Indonesia: an optimized spatial clustering approach” [4]. The study analyzed 511 districts that came from the calibration with the geographic map of Indonesia in 2013. The calibration was used because, the number of districs were different between two years (511 districts in 2013 and 514 districts in 2017). As a result, it was essential to adjust the 2017 districts into the 2013 districts to obtain spatial information. Based on the expansion history, the districts need to be adjusted were Buton (now South Buton and Central Buton) and Muna (now Muna and West Muna). The primary data source used in the research was the 2017 National Socio-Economic Survey (SUSENAS) [6]. Meanwhile, population and growth data were obtained from Indonesia's population projection in 2017 [7]. Table 1, and Table 2 shows the description of the variables in the dataset and the sample of the data from seven districts respectively.

**Table 1:** Variable description

Label	Variable	Description
DISTRICTCODE	District Code	Code of the region/district
CHILDREN	Children	Percentage of under five years old population
FEMALE	Female	Percentage of female population
ELDERLY	Elderly	Percentage of 65 years old and overpopulation
FHEAD	Female household	Percentage of households with female head of household
FAMILYSIZE	Household members	The average number of household members in one district
NOELECTRIC	Non-electric household	Percentage of households that do not use electricity as lighting sources
LOWEDU	Low education	Percentage of 15 years and overpopulation with low education
GROWTH	Population growth	Percentage of population change
POVERTY	Poverty	Percentage of poor people
ILLITERATE	Illiteracy	Percentage of population that cannot read and write
NOTRAINING	Training	Percentage of households that did not get disaster training
DPRONE	Disaster prone	Percentage of households living in disaster-prone areas
RENTED	Homeownership	Percentage of households renting a house
NOSEWER	Drainage	Percentage of households that did not have a drainage system
TAPWATER	Water source	Percentage of households that use piped water
POPULATION	Population	Number of Population

**Table 2:** Sample Data of social vulnerability

DISTRICT CODE	CHILDREN	FEMALE	ELDERLY	FHEAD	FAMILY SIZE	NO ELECTRIC	LOWEDU	GROWTH
1101	8.00	48.78	2.18	13.11	4.06	1.43	25.65	1.25
1102	13.52	49.69	2.30	13.17	4.48	1.07	28.72	2.29
1103	9.44	50.78	4.90	20.74	4.24	0.50	29.78	1.52
1104	11.19	50.10	2.74	17.78	4.25	2.02	16.79	2.11
1105	11.68	50.05	2.76	19.47	4.30	0.60	32.84	2.02
1106	11.31	49.77	2.96	12.68	3.74	0.00	22.41	2.03
1107	10.24	49.46	3.38	17.04	4.07	2.30	26.01	2.02


POVERTY	ILLITERATE	NOTRAINING	DPRONE	RENTED	NOSEWER	TAPWATER	POPULATION	
20.20	5.02	92.72	48.81	4.88	22.89	5.60	91372	
22.11	10.98	97.90	73.09	6.68	20.01	13.40	119490	
14.07	7.72	98.76	77.14	3.34	31.79	6.98	231893	
14.86	6.67	99.88	94.28	4.05	43.55	20.30	208481	
15.25	6.65	99.76	82.24	2.32	26.79	12.99	419594	
16.84	4.78	97.66	85.70	8.96	16.36	12.69	204273	
20.28	5.46	93.66	36.27	8.10	23.31	9.36	201682	

Other than the social vulnerability analysis, the data can be used for many purposes. For example, it can be elaborated to analyze the development condition in Indonesia in districts level. The analysis could offer a deeper understanding of the condition of Indonesia in a specific manner. Moreover, the dataset can also be used to identify the priority areas based on the available indicators, particularly, the social vulnerability. The characteristic of districts in Indonesia tends to be different so that it is necessary to make a deeper analysis for policymaking. Subsequently, the distance matrix could be harnessed to perform spatial analysis to investigate the interregional development. Lastly, this dataset can be combined with the dataset from different fields to create a well-crafted and deeper multidisciplinary analysis, particularly the social vulnerability in other sectors' contexts.

## 2 Materials and Methods

### 2.1 Brief information about SUSENAS

National socioeconomic survey (SUSENAS) is a survey conducted by BPS-Statistics Indonesia to collect the primary data about household's welfare from social and economic characteristics. The data was collected by interviewing the selected households directly with multi-stage sampling (see [8]) for details. Many crucial indicators are estimated based on data from SUSENAS, mainly per capita expenditure, poverty, and Gini ratio. Other indicators such as education, health, and demographic characteristics are also calculated based on the data from this survey. The estimation are usually done annually at the district level. As a result, the data is useful as the basis of national and regional development and planning.

To obtain the social vulnerability-related variables, we selected related information based on the SUSENAS questionnaire (see [6] for more details). The variables and associated questions can be seen in table 3. There were three components in estimating the SUSENAS' indicators: the region, relevant data, and weight. The data aggregation was done by utilizing the weight to do a cross-tabulation between the areas and the data. This study used the *dplyr* [10] and *descr* [11] package to transform and cross-tabulate the data from its raw form, respectively.

**Table 3:** Source of variables

Label	Question details	Question
DISTRICTCODE	R101 and R102	Province and District
CHILDREN	R407< 5 years	How old is (name)?
FEMALE	R405	Is (name) male or female
ELDERLY	R407>65 years	How old is (name)?
FHEAD	R403 and R405	What is the relation with the head of household?
FAMILYSIZE	R301	Number of family members
NOELECTRIC	R1618	What is the primary source of lighting in this house?
LOWEDU	R514 and R517	R514: What is the highest education which is currently/has been done by (name) R517: What is the highest education certification obtained by (name)?
GROWTH	-	BPS-Statistics Indonesia's population projection [7]
POVERTY	-	Compiled in BPS-Statistics Indonesia website [9]
ILLITERATE	R511-R513	Is (name) can read and write a simple sentence using Latin/Arabic/others letters?
NOTRAINING	R1804	In one last year, has anyone in (name) 's household ever participate in disaster training/simulation?
DPRONE	R1802A	In one last year, has anyone in (name) 's household ever occurred any natural disaster (e.g., earthquake, flood, tsunami, cyclone)?
RENTED	R1602	What is the ownership status of the current house
NOSEWER	R1610A	Does it have an excellent waste facility, and who is using that?
TAPWATER	R1611A and R1616A	R1611A: What is the water source of drink in this household? R1616A: What is the water source of cooking/bathing/washing in this household?
POPULATION	-	Compiled in BPS-Statistics Indonesia and Indonesia Population projection publication

**Fig. 1:**
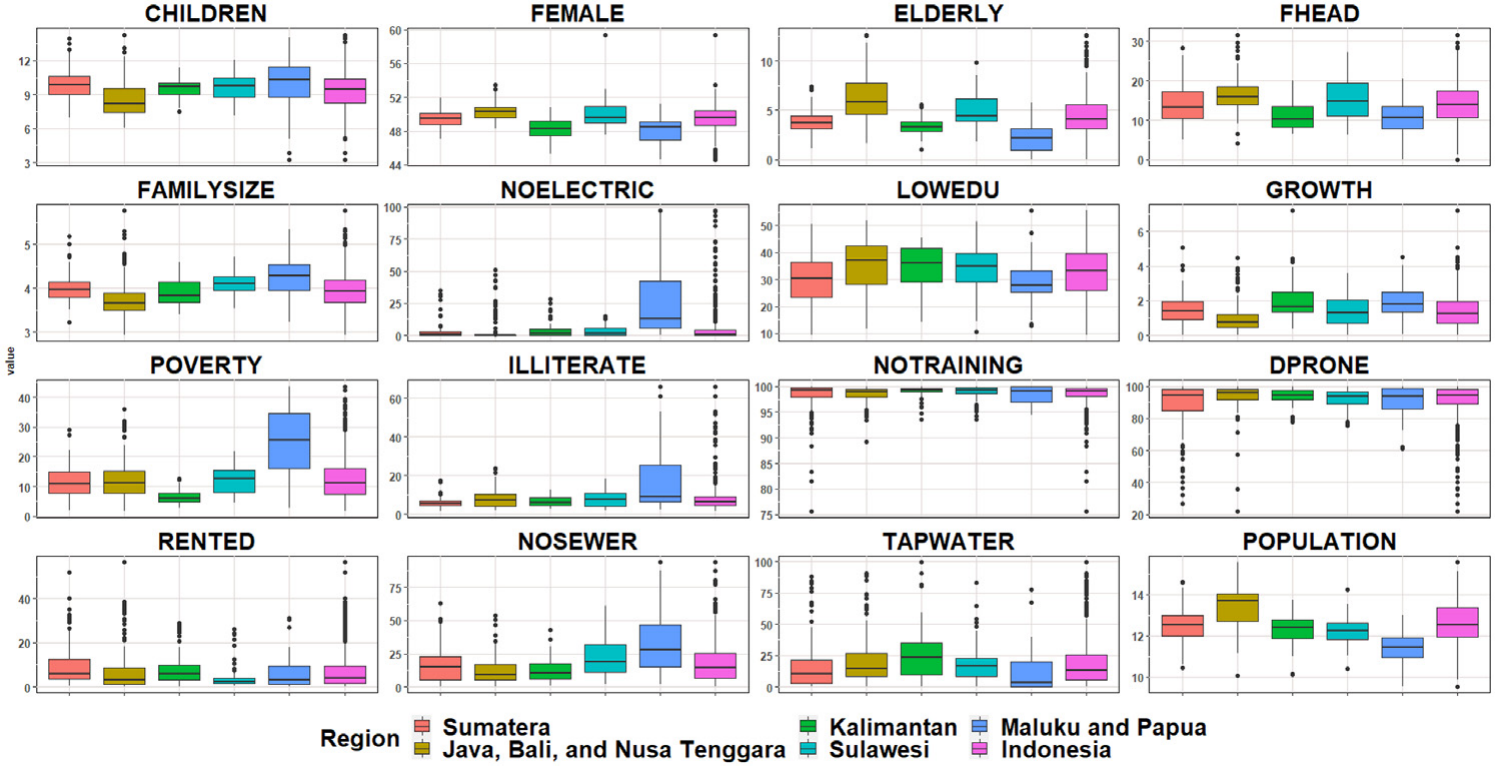
Boxplot of social vulnerability characteristics based on region

### 2.2 Distance matrix formation

The distance matrix in this study was constructed from a geographic map of Indonesia in 2013. The map format was in shapefile – a file format which stored the geometric location and geospatial information. Consequently, we needed to pre-process the file into numerical form to construct the distance matrix. The distance matrix was also constructed using R, specifically the package *rgeos, rgdal*, and *sp* [12], [13]. First, we read the shapefile using the *readOGR* function, followed by *gcentroid* to obtain each district's center coordinate along with its region code. Subsequently, the distance matrix was calculated using *spDists* function which returns the distance in kilometres. Then, we matched the districts’ code from the map to the districts’ code in SUSENAS data. Due to the difference in the number of districts, the unmatched district in SUSENAS data were joined with its parent district in 2013. Finally, the distance matrix were matched with the available district code.

## 3 Data Condition

Figure 1 shows the distribution of social vulnerability characteristics in Indonesia in 2017. Boxplot was used to assess the distribution of data. In Figure 1, all variables are distributed in percentage, except the population that is distributed using logs to simplify data distribution. The colors in the figure represent the available areas in the plot legend. The pink box plot represents the distribution of national-level data in Indonesia.

Based on Figure 1, it can be seen that almost all variables have outliers. It indicates that there was inequality among regions in Indonesia in the context of social vulnerability. It was supported by the results' details, which disseminate different interregional characteristics such as demography. Eastern Indonesia, namely Maluku and Papua, tend to have a low female and elderly population. In contrary, the children and family size in these regions tend to be higher (also with relatively high dispersion) than the other regions. Indonesia had an asymmetrical distribution of the population (in log, while the growth dispersion was asymmetric from the population aspects. The Java, Bali, and Nusa Tenggara region had smaller population growth due to the large population in each district. Moreover, the same pattern was followed by the rest of the regions.

Maluku and Papua had the most problems among the other regions in Indonesia. The non-electricity, poverty, illiteracy, and non-sewer variables, in these two areas were higher compared to those variables in the other regions. All regions tend to have high percentage of households with no disaster training. Unfortunately, most of these regions were also those that were prone to disaster. On the other hand, some regions had lower percentages of disaster-prone households, which considered outliers (Sumatra and Java, Bali, and the Nusa Tenggara region). Regarding the housing, the Sumatra region had the highest percentage of people renting houses.

## Data Availability

The data can also be accessed directly in https://raw.githubusercontent.com/bmlmcmc/naspaclust/main/data/sovi_data.csv for social vulnerability data and https://raw.githubusercontent.com/bmlmcmc/naspaclust/main/data/distance.csv for the distance matrix.

## Ethics Statement

There is no conflict of interest. The data is available in public domain.

## CRediT Author Statement

**Robert Kurniawan:** Conceptualization, Methodology, Writing-Reviewing, and Editing**. Bahrul Ilmi Nasution**: Data curation, Visualization, Writing-original draft preparation. **Neli Agustina**: Editing and Investigation. **Budi Yuniarto:** Data curation and Visualization**.**

## Declaration of Competing Interest

The authors declare that they have no known competing financial interests or personal relationships which have or could be perceived to have influenced the work reported in this article.
